# Supplementary material for: Diversification of division mechanisms in endospore-forming bacteria revealed by analyses of peptidoglycan synthesis in Clostridioides difficile
Source: Nat Commun. 2023 Dec 2;14:7975. doi: 10.1038/s41467-023-43595-3 (PMC10693644; doi:10.1038/s41467-023-43595-3)
Supplement: Supplementary file 3 — Description of Additional Supplementary Files [file 41467_2023_43595_MOESM3_ESM.pdf]

Description of Additional Supplementary Files:

Supplementary Data 1: Genome accession numbers and coordinates corresponding to dcw clusters shown in Figure 1.

Supplementary Data 2: Accession numbers of the sequences and summary data used to infer the information presented in Fig. 1, Supplementary Fig. 1-2, and Supplementary Data 3.

Supplementary Data 3: Jaccard similarity coefficients between sets of organisms grouped according to presence/absence of dcw SEDS (spoVE/ftsW) and sporulation (spo0A & spoIIE) genes.

Supplementary Data 4: C. difficile strains used in this study.

Supplementary Data 5: E. coli strains used in this study.

Supplementary Data 6: RT-qPCR primers used for gene expression analyses shown in Supplementary Fig. 6.
