# Supplementary material for: Diversification of division mechanisms in endospore-forming bacteria revealed by analyses of peptidoglycan synthesis in Clostridioides difficile
Source: Nat Commun. 2023 Dec 2;14:7975. doi: 10.1038/s41467-023-43595-3 (PMC10693644; doi:10.1038/s41467-023-43595-3)
Supplement: Supplementary file 5 — Reporting Summary [file 41467_2023_43595_MOESM5_ESM.pdf]

nature portfolio

Corresponding author(s): Aimee Shen
Last updated by author(s): 11/10/23

# Reporting Summary

Nature Portfolio wishes to improve the reproducibility of the work that we publish. This form provides structure for consistency and transparency in reporting. For further information on Nature Portfolio policies, see our Editorial Policies and the Editorial Policy Checklist.

## Statistics

For all statistical analyses, confirm that the following items are present in the figure legend, table legend, main text, or Methods section.

| n/a | Confirmed | |
|---|---|---|
| ☐ | ☒ | The exact sample size (*n*) for each experimental group/condition, given as a discrete number and unit of measurement |
| ☐ | ☒ | A statement on whether measurements were taken from distinct samples or whether the same sample was measured repeatedly |
| ☐ | ☒ | The statistical test(s) used AND whether they are one- or two-sided<br>*Only common tests should be described solely by name; describe more complex techniques in the Methods section.* |
| ☒ | ☐ | A description of all covariates tested |
| ☒ | ☐ | A description of any assumptions or corrections, such as tests of normality and adjustment for multiple comparisons |
| ☐ | ☒ | A full description of the statistical parameters including central tendency (e.g. means) or other basic estimates (e.g. regression coefficient) AND variation (e.g. standard deviation) or associated estimates of uncertainty (e.g. confidence intervals) |
| ☐ | ☒ | For null hypothesis testing, the test statistic (e.g. $F$, $t$, $r$) with confidence intervals, effect sizes, degrees of freedom and $P$ value noted<br>*Give P values as exact values whenever suitable.* |
| ☒ | ☐ | For Bayesian analysis, information on the choice of priors and Markov chain Monte Carlo settings |
| ☒ | ☐ | For hierarchical and complex designs, identification of the appropriate level for tests and full reporting of outcomes |
| ☒ | ☐ | Estimates of effect sizes (e.g. Cohen's $d$, Pearson's $r$), indicating how they were calculated |

*Our web collection on statistics for biologists contains articles on many of the points above.*

## Software and code

Policy information about availability of computer code

| | |
|---|---|
| Data collection | n/a |
| Data analysis | HMMER-3.1b2 package, MAFFT-v7.407, MAFFT v7.481, BMGE-1.1, IQTREE-1.6.3, IQ-TREE v2.0.7, trimAl 1.2rev59, MacSyFinder2, IToL, RStudio 1.4.1717, Clinker; Databases: Pfam 29.0 |

For manuscripts utilizing custom algorithms or software that are central to the research but not yet described in published literature, software must be made available to editors and reviewers. We strongly encourage code deposition in a community repository (e.g. GitHub). See the Nature Portfolio guidelines for submitting code & software for further information.

## Data

Policy information about availability of data

All manuscripts must include a data availability statement. This statement should provide the following information, where applicable:

- Accession codes, unique identifiers, or web links for publicly available datasets
- A description of any restrictions on data availability
- For clinical datasets or third party data, please ensure that the statement adheres to our policy

*Provide your data availability statement here.*

## Research involving human participants, their data, or biological material

Policy information about studies with human participants or human data. See also policy information about sex, gender (identity/presentation), and sexual orientation and race, ethnicity and racism.

| | |
|---|---|
| Reporting on sex and gender | n/a |
| Reporting on race, ethnicity, or other socially relevant groupings | n/a |
| Population characteristics | n/a |
| Recruitment | n/a |
| Ethics oversight | n/a |

Note that full information on the approval of the study protocol must also be provided in the manuscript.

# Field-specific reporting

Please select the one below that is the best fit for your research. If you are not sure, read the appropriate sections before making your selection.

☒ Life sciences ☐ Behavioural & social sciences ☐ Ecological, evolutionary & environmental sciences

For a reference copy of the document with all sections, see nature.com/documents/nr-reporting-summary-flat.pdf

# Life sciences study design

All studies must disclose on these points even when the disclosure is negative.

| | |
|---|---|
| Sample size | Sample sizes for image quantification were based on individual cell quantifications of multiple fields of view across multiple replicates. A minimum of 100 sporulating cells within a given replicate were quantified based on prior studies. This number ensured that ≥10 cells in each sporulation stage could be observed in WT for each replicate. For other cell measurements, thousands of cells were quantified across multiple replicates based on the number of cells in a given field of view for multiple fields of view across a given replicate. |
| Data exclusions | no data was excluded |
| Replication | Multiple independent experiments were conducted for all data shown, with ≥ three biological replicates were performed for most experiments. |
| Randomization | Mutants were compared to wild-type or complementation strains. |
| Blinding | Blinding was not relevant to our study because morphological differences were easily discerned for mutants where differences are reported, rendering blinding irrelevant. |

# Reporting for specific materials, systems and methods

We require information from authors about some types of materials, experimental systems and methods used in many studies. Here, indicate whether each material, system or method listed is relevant to your study. If you are not sure if a list item applies to your research, read the appropriate section before selecting a response.

### Materials & experimental systems

| n/a | Involved in the study | |
|---|---|---|
| ☐ | ☒ | Antibodies |
| ☒ | ☐ | Eukaryotic cell lines |
| ☒ | ☐ | Palaeontology and archaeology |
| ☒ | ☐ | Animals and other organisms |
| ☒ | ☐ | Clinical data |
| ☒ | ☐ | Dual use research of concern |
| ☒ | ☐ | Plants |

### Methods

| n/a | Involved in the study | |
|---|---|---|
| ☒ | ☐ | ChIP-seq |
| ☒ | ☐ | Flow cytometry |
| ☒ | ☐ | MRI-based neuroimaging |

## Antibodies

| | |
|---|---|
| Antibodies used | No commercial primary antibodies were used in the study. All antibodies against C. difficile spore proteins were generated by Cocalico Biologicals as described. Secondary antibodies were from LiCor Biosciences (926-32211, 926-68070). Primary antibodies were used at between 1:1,000 and 1:3,000 dilution (details provided in the Methods section). Secondary antibodies were used at 1:20,000 dilution. |

| | |
|---|---|
| Validation | The specificity of all antibodies used was determined by including a mutant lacking the protein being detected as a negative control. |

## Plants

| | |
|---|---|
| Seed stocks | n/a |
| Novel plant genotypes | n/a |
| Authentication | n/a |
